# U.S. Consumers’ Tree Nut Food Safety Knowledge, Perceptions, and Handling Practices across Demographic Groups

**DOI:** 10.3390/foods12234289

**Published:** 2023-11-28

**Authors:** Maeve Riley Swinehart, Yaohua Feng

**Affiliations:** Department of Food Science, Purdue University, 745 Agriculture Mall Drive, West Lafayette, IN 47907, USA

**Keywords:** consumer survey, food safety education, soaked nuts, nut-based dairy analogs

## Abstract

Tree nuts are often perceived as presenting a low risk for foodborne illness, despite their association with several foodborne outbreaks and recalls in recent years. An online survey was designed to assess how consumers’ food safety knowledge, perception of risks and benefits, and preferred sources for food safety information influence their tree nut handling behavior. Participants (*n* = 981) who soaked tree nuts or prepared nut-based dairy analogs (NBDAs) at home completed the survey. Their responses indicated insufficient knowledge about potential contaminations of tree nuts. Only 25% of participants had heard of a tree nut-related outbreak or recall. Few (30%) participants perceived a risk of contracting a foodborne illness from tree nuts. The participants were more concerned with the health benefits than potential microbial risks of raw tree nuts and preferred government agencies for tree nut food safety information. Based on a cluster analysis, demographics with lower food safety knowledge and risk perceptions (ages 18–24 or 45 and above, female, suburban and rural communities, have less than a bachelor’s degree, and earned less than USD 100,000 annually) tended to engage in risky tree nut handling practices (*p* < 0.05). The findings of this study support the development of audience-targeted food safety extension materials for tree nuts.

## 1. Introduction

The consumption of tree nuts and products made from them has increased in recent years [1], in part due to their purported nutritional value and health benefits [2,3,4,5,6,7,8]. As a low-moisture food (water activity (aw) of <0.85), tree nuts do not provide sufficient moisture to support pathogen growth, but some foodborne pathogens such as Salmonella have developed the ability to survive at lower water activities for long periods of time [9,10]. Additionally, some consumers may add water to soak tree nuts or make nut-based dairy analogs (NBDAs) [7,8,11]. Recent studies examining soaked almonds and walnuts reported that foodborne pathogens, including Salmonella, Escherichia coli, and Listeria monocytogenes, significantly increase within 24 h of soaking at ambient temperature (23 °C) [7,8]. However, little is known about the fate of foodborne pathogens during the handling of tree nuts.

Tree nuts can be contaminated at multiple points from harvest to consumption. Previous studies isolated Salmonella from raw cashews, macadamia nuts, hazelnuts, pine nuts, pecans, walnuts, and pistachios sold for retail in the U.S. [12,13]. Raw tree nuts were associated with multiple outbreaks and recalls, including products made from them [14]. And, while commercial almonds require pasteurization, other tree nuts are not upheld to the same standard [15].

Consumers’ food safety knowledge and their perceptions of food-associated risks can influence their food handling practices in the home [16,17,18,19]. A risk perception is an individual or group judgment of the magnitude and likelihood of a negative outcome from an action [20]. In general, consumers perceive low-moisture foods, such as tree nuts, as presenting a low risk for foodborne illnesses [21,22,23] and believe that tree nuts have many health benefits [24].

Several behavior change models have been used to explain the relationships between knowledge, perceptions, and behavior. The health belief model (HBM) attempts to explain people’s behaviors related to health protection [25]. The application of the HBM in food safety has indicated that individuals who perceive the potential for a personal health treatment from food are more likely to engage in food safety behavior [26]. The theory of planned behavior (TPB) aims to predict the intention of individuals to perform a given behavior based on their attitudes, subjective norms, and perceived behavior control [27]. The TPB has been successful in explaining food safety behavior via risk perceptions [28]. Both the HBM and TBP may be used to encourage positive behavior change when handling tree nuts at home.

Several studies reveal that knowledge, risk perception, and behaviors vary among demographic groups [17,19,29]. The knowledge, perceptions, and handling practices of individuals may be influenced by their age, gender, ethnicity, community, educational background, and/or income. For example, young adults tend to have lower food safety knowledge, risk perceptions, and safe handling practices than older adults due to reliance on family and friends for food safety information [16,30]. Similarly, males tend to have less food preparation experience than females and engage in food handling practices that are less safe [16,17,18,31].

Additionally, the sources of information can affect consumers’ knowledge, risk perceptions, and handling behaviors [29]. Increased exposure to food safety incidents can heighten their risk perceptions and safe food handling practices [32]. Also, the level of trust that consumers have for the information source can drive their behavior change [33]. Thus, understanding tree nut consumers’ knowledge, perceptions, and trusted sources can help promote safe handling practices.

At the time of this study, limited data were available on consumers’ food safety knowledge, perceptions of risks and benefits, and preferred sources for food safety information on tree nuts and products made from them, such as soaked nuts and NBDAs. The present study aims to address this data gap through an online survey to help develop audience-tailored educational materials on tree nuts to promote safe handling practices and prevent foodborne illnesses.

## 2. Materials and Methods

### 2.1. Pilot Study

The research protocol (IRB-2019-6669) was approved by the Institutional Review Board (IRB) at Purdue University (West Lafayette, IN, USA). The questions were developed by the authors to address the objectives of the present study and were modeled after previous consumer surveys [7,21,32,34]. The survey was reviewed by three food safety experts for accuracy and to ensure that the questions effectively captured the study objectives. It was then distributed to a convenience sample of 12 consumers of tree nuts to assess the clarity and comprehensibility of each question during a guided reading of the survey conducted by the main researcher. Next, the survey was pilot tested in December 2021 using a consumer panel generated by Qualtrics XM (Qualtrics Software Company, Seattle, WA, USA), an external online survey company. Participant selection criteria included U.S. consumers who (a) were 18 years or older, (b) were the primary grocery shopper for the household, (c) had prepared soaked tree nuts for direct consumption or production of NBDAs (milk, cheese, and yogurt only) at least two times in the past year, and (d) used almonds, cashews, pistachios, or walnuts. The pilot test included 50 participants who qualified and completed the online survey. The results highlighted inconsistencies or errors within the survey that were then modified to improve the accuracy of the survey. The revised survey was used for the full study.

### 2.2. Participants

Participants were recruited in January 2022 through Qualtrics XM (Qualtrics Software Company). Participants were selected based on the same inclusion criteria mentioned above for the pilot test, permitting only consumers who soaked tree nuts and/or prepared NBDAs two or more times a year. The participants were required to answer every question. The average time for the participants to complete the survey was 5 min.

### 2.3. Survey Questions

**Figure 1:**
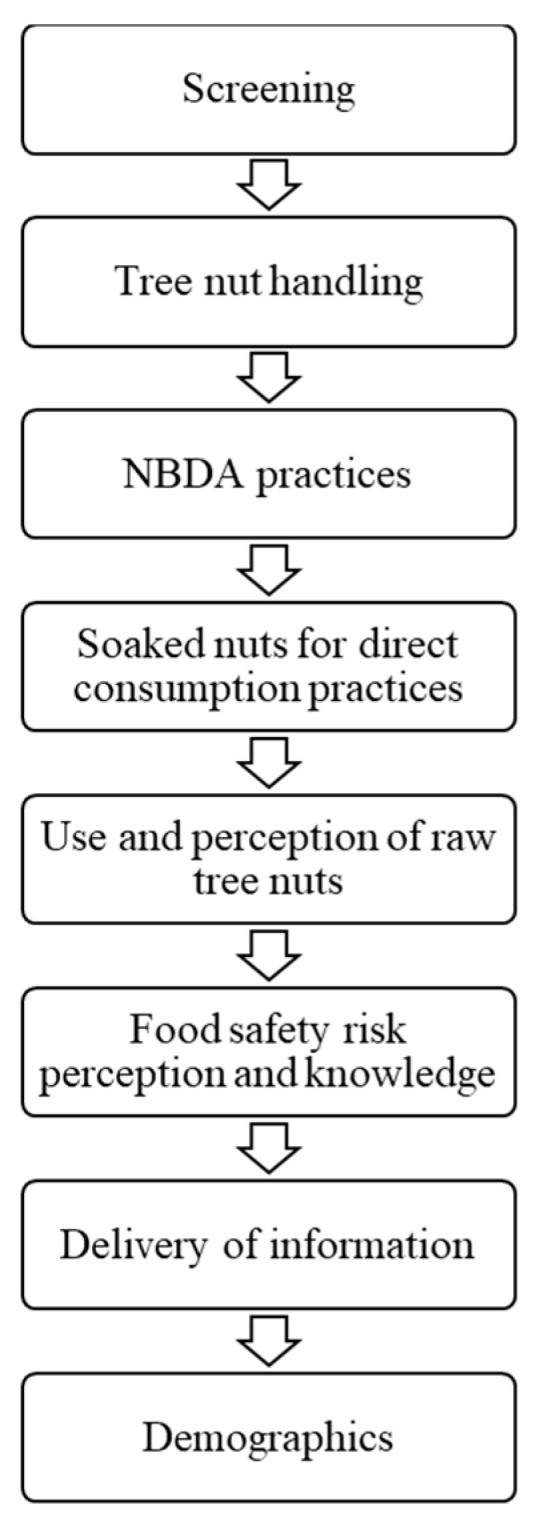
General flow of survey questions.

The general flow of the survey questions is illustrated in Figure 1. The survey questions focused on four types of tree nuts—almonds, cashews, pistachios, and walnuts. The survey consisted of 107 multiple choice, true or false, and Likert scale-ranking questions that included the screening questions, and that covered tree nut handling, use of raw tree nuts, food safety knowledge, and perceptions associated with the preparation and consumption of tree nuts. If participants had soaked nuts for direct consumption or made NBDAs two or more times in the past year with any of the four nut varieties, they were presented with a series of questions pertaining to their handling practices, knowledge, and risk perceptions. Cronbach’s alpha for internal consistency was calculated (α = 0.77). Cronbach alpha was calculated on a scale of 0 to 1, where a higher Cronbach alpha value indicates a greater reliability of consistent responses from each participant among a set of questions. All survey questions can be found in Supplementary Materials. The present study reports survey data on the reasons participants chose to make NBDAs at home (4 questions), their knowledge and perceptions of tree nut food safety (19 questions), and preference for educational materials (3 questions). The survey responses to questions pertaining to the handling and use of tree nuts in the home, with a focus on the preparation of soaked nuts and NBDAs, were reported in a separate manuscript [35].

### 2.4. Screening and Demographic Questions

The participants were provided definitions of tree nuts, soaking, and NBDAs at the beginning of the survey. The participants were then presented with four screening questions, beginning with “Are you the primary grocery shopper for your household?” Those who answered “no” were excluded from the survey. The remaining participants were then asked, “How often do you soak tree nuts for direct consumption?” and “How often do you use tree nuts to make NBDA?” Those who answered “never” or “rarely” on both questions were excluded from the survey. The participants were asked to select which of the following tree nuts they soak for direct consumption and to make NBDAs: almonds, cashews, peanuts, pecans, pistachios, and walnuts. Survey participants who selected only peanuts and pecans (four in total) were excluded from that point forward because these nuts are not as widely soaked for direct consumption and/or to make NBDAs when compared to the other four nuts [11]. Seven demographic questions were asked at the end of the survey to collect data on age, gender, ethnicity, residency, educational background, and household income (Table 1).

### 2.5. Tree Nut Knowledge and Perception Questions

The participants were asked why they chose to soak nuts and/or make NBDAs at home rather than buying (a) commercially processed nuts, and (b) dairy-based products. Then, a series of questions addressed the participants’ tree nut knowledge and perceptions. Knowledge questions were marked as correct or incorrect with “Does not know” responses recorded as incorrect. The perception questions asked participants about their perception of raw and treated tree nuts and the risks associated with the preparation of tree nuts, and about the health benefits of preparing soaked nuts and NBDAs at home. The responses to the risk perception questions were scored using a 5-point Likert scale from 1 (“Strongly disagree”) to 5 (“Strongly agree”); and the mean scores and standard deviations were calculated. The participants were asked what their response would be to a tree nut-related foodborne outbreak or recall. Participants were then asked how strongly entities in the food supply chain—the farmers, processors, supermarkets, food companies, and government—were responsible for a tree nut outbreak, and how much they would continue to trust each entity following the outbreak. Lastly, the participants were asked to identify their preferred source for tree nut food safety information.

### 2.6. Data Analysis

The survey responses were organized into a Microsoft Excel 2019 spreadsheet (Microsoft Corporation, Redmond, WA, USA) to conduct a descriptive analysis of the data. Microsoft Excel 2019 calculated the frequency of responses for each answer option for every question.

A two-step cluster analysis with log-likelihood measures was conducted using IBM SPSS Statistics 28.2.2 for Windows (IBM Japan, Ltd., Tokyo, Japan) to identify the food safety knowledge, perceptions, and handling practices of tree nut consumers across different demographic groups. A Chi-square test was performed for each variable to indicate significant differences among the clusters. One-way ANOVAs were conducted to determine significant differences for the mean scores of knowledge and perception statements between the two clusters (Welch’s F are reported due to non-homogeneity of variances). The differences were considered significant when a *p*-value of <0.05 was found.

To evaluate the correlation between participants’ tree nut knowledge, risk perceptions, and handling practices, the Pearson correlation coefficients were calculated using SPSS version 26. A Pearson correlation coefficient equal to or exceeding 0.2 was used to indicate an adequate correlation between two variables [36]. Two-sample *t*-tests were performed to indicate the significant differences between two variables. The differences were considered significant when a *p*-value of <0.05 was found.

## 3. Results

Among a total of 1532 consumers who attempted the survey, 981 (64%) met all three inclusion criteria and were included in the analysis. Most of the participants were between 25 and 44 years of age (58%; *n* = 981); female (62%; *n* = 981); white, non-Hispanic (67%; *n* = 981); lived in a suburban area (43%; *n* = 981) held a bachelor’s or higher advanced degree (43%; *n* = 981); and earned an annual household income of less than USD 75,000 (60%; *n* = 981) (Table 1). The participants reported that they obtained recipes for soaking nuts for direct consumption most often from YouTube (59%; *n* = 655), social media (52%; *n* = 655), and cookbooks (37%; *n* = 655), and recipes for NBDAs most frequently from YouTube (59%; *n* = 934), cookbooks (41%; *n* = 934), and family members and friends (33%; *n* = 934) (Supplemental Table S1). Some participants selected more than one source for recipes for soaked nuts (*n* = 420) and NBDAs (*n* = 610).

### 3.1. Knowledge of Tree Nuts

**Figure 2:**
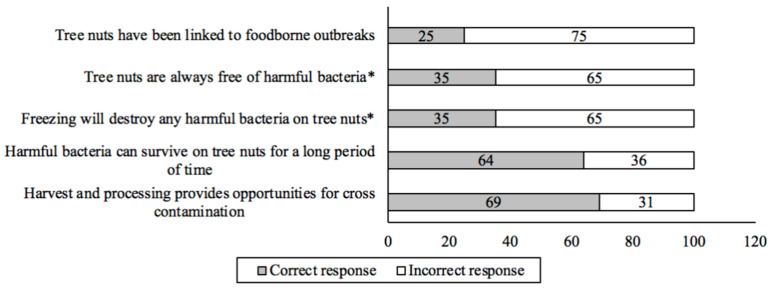
Participants’ (*n* = 981) food safety knowledge of tree nuts (* = incorrect reverse statements).

When asked how knowledgeable they were about how tree nuts were grown and harvested, 69% (*n* = 981) considered themselves to be somewhat or very knowledgeable (Table S2), and 69% (*n* = 981) knew that harvesting and processing tree nuts provides opportunities for cross contamination (Figure 2). However, nearly half (46%; *n* = 981) of the participants were surprised to learn that tree nuts are left on the ground to dry (Table S3).

When asked about their food safety knowledge, a total of 247 participants (25%) were aware of foodborne outbreaks or recalls associated with tree nuts (Figure 2). Of those 247 participants, 50% had heard of outbreaks or recalls involving walnuts, 49% for almonds, 46% for cashews, and 34% for pistachios (Table S4). About two thirds (64%; *n* = 981) of the participants acknowledged that harmful bacteria could survive on tree nuts for a long period of time, 35% (*n* = 981) indicated that the tree nuts they purchase may be contaminated with harmful bacteria, and 35% (*n* = 981) of participants were aware that freezing would not kill any harmful pathogens present on the tree nuts (Figure 2). The food safety contaminations about which participants were most concerned were from dust and other particles (64%; *n* = 981) (Table S3).

### 3.2. Perceptions of Food Safety Risks

**Figure 3:**
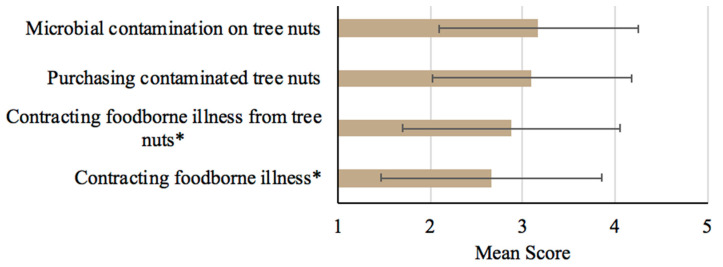
Means and standard deviations of participants’ risk perceptions of tree nuts (range: 1 = very low risk to 5 = very high risk; * *p*-value < 0.001).

The participants’ food safety risk perceptions of tree nuts were relatively low. Only 24% (*n* = 981) of participants perceived that tree nuts may place them at a high risk of contracting a foodborne illness, while 30% (*n* = 981) felt they were likely to contract a foodborne illness from consuming tree nuts (Table S5). The perceived risk of contracting a foodborne illness from consuming tree nuts (mean = 2.87, standard deviation = 1.10) was statistically higher (*p* < 0.001) than the perceived risk of contracting a foodborne illness in general (mean = 2.66, standard deviation = 1.20) (Figure 3). More than a third (37%; *n* = 981) indicated that the tree nuts they purchase are likely to be contaminated (mean = 3.10, standard deviation = 1.08), and 39% (*n* = 981) believed that tree nuts pose a high risk for microbial contamination (mean = 3.17, standard deviation = 1.08) (Table S5; Figure 3).

### 3.3. Perceptions of Raw and Pasteurized Tree Nuts

**Figure 4:**
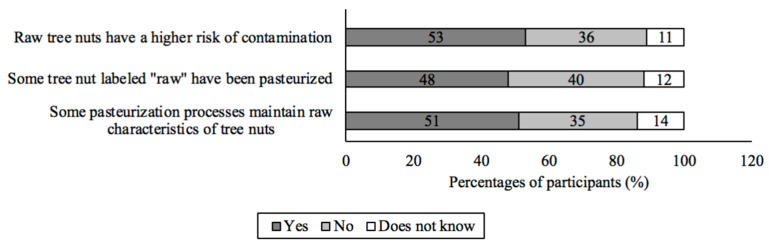
Participants’ perceptions of raw and pasteurized tree nuts.

More than half (53%; *n* = 981) of participants perceived that raw tree nuts present an increased risk for contamination, 48% believed that some tree nuts labeled “raw” have been pasteurized, and 51% recognized that pasteurized tree nuts maintain their raw characteristics (Figure 4). But when asked what raw characteristics they believed to be lost during pasteurization, only 15% (*n* = 981) indicated that no characteristics were lost (Table S6). The participants believed that the most common raw characteristics lost during pasteurization are the flavor (39%; *n* = 981); unsaturated fats, vitamins, and minerals (37%; *n* = 981); high protein and dietary fiber content (37%; *n* = 981); and freshness (37%; *n* = 981). When using tree nuts at home, 323 (33%) participants preferred to use raw tree nuts in contrast to treated (pasteurized, blanched, or roasted) tree nuts (Table S7). The participants reported preferring raw over treated tree nuts because raw tree nuts are “free of preservatives, additives, or chemical substances” (47%; *n* = 323) and had “nutrients intact” (40%; *n* = 323).

The participants evaluated two statements about raw tree nuts before and after reading a food safety message on the risk of foodborne pathogen survival on tree nuts (Table 2). The message was: “Pathogens, such as Salmonella, can survive for sufficient time on low-moisture foods, such as tree nuts, to cause foodborne illness. Salmonella infections can lead to diarrhea, fever, and abdominal cramps and for the immunocompromised, elderly, and young children can cause a much more serious infection.” After reading that message, significantly more participants disagreed with the following statement: “raw tree nuts have more of a health benefit than treated tree nuts” (*p* < 0.001 from Chi-square test). Similarly, significantly more participants also disagreed with the following statement: “the health benefits of raw tree nuts are more important than the microbial risk” (*p* < 0.001 from Chi-square test).

### 3.4. Perceived Health Benefits of Soaking Nuts and Homemade NBDA

Of the 981 participants that included in the survey, 655 (67%) soaked nuts in water for direct consumption, and 934 (95%) prepared NBDAs at home. The participants who soaked tree nuts at home for direct consumption and/or for preparation of NBDAs (*n* = 718) believed that there are many benefits to the practice of soaking nuts (Table S8). The common benefits reported were that soaking “removes chemical substances such as pesticides, preservatives, and additives” (49%; *n* = 718), “removes harmful bacteria” (48%; *n* = 718), “removes nutritional inhibitors such as phytic acid and tannin” (38%; *n* = 718), and “aids in digestion of nutrients” (31%; *n* = 718).

Of the participants who chose to prepare NBDAs (*n* = 934), a plethora of reasons were provided for processing at home rather than buying commercially, including for health (52%), freshness (42%), flavor (35%), and being free of preservatives or additives (32%) (Table S9). Similarly, the most common response for why participants make NBDAs rather than dairy-based products at home was for health (61%; *n* = 718) (Table S8). The main method of consuming homemade NBDAs was “directly as it is” (56%; *n* = 934), “added to cereals or oatmeal” (52%; *n* = 934), or “in baked products” (46%; *n* = 934) (Table S10).

### 3.5. Response to Outbreaks and Recalls

When participants were asked what actions they would take if the tree nuts they had purchased were recalled, most (75%; *n* = 981) reported that they would throw away the recalled nuts (Table S11). However, 13% (*n* = 981) of participants would cook the recalled nuts, 11% (*n* = 981) would continue to eat them, and 10% (*n* = 981) would continue to handle them to feed their pets or use for crafts.

**Figure 5:**
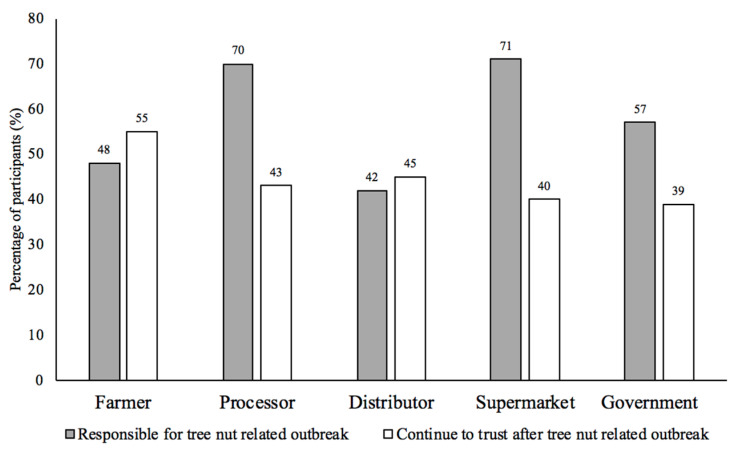
Participants’ (n = 981) belief in who is responsible for a tree nut-related outbreak and who they would continue to trust after a tree nut-related outbreak. Due to some participants’ selecting more than one entity, the calculated percentages exceed 100.

**Figure 6:**
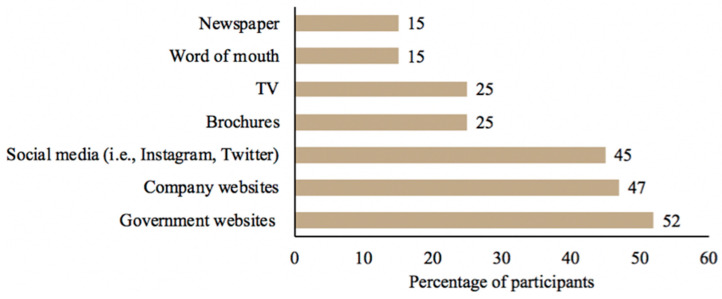
Participants’ (*n* = 981) preferred sources for tree nut food safety information.

Figure 5 depicts the entities in the food supply chain that participants believed were responsible and trustworthy during a tree nut-related foodborne outbreak or recall. The participants most often reported that the supermarkets (71%; *n* = 981) and processors (70%; *n* = 981) were responsible. Meanwhile, participants would continue to trust farmers (55%; *n* = 981) and distributors (45%; *n* = 981) the most following a tree nut-related outbreak or recall. The participants most commonly (56%; *n* = 981) reported that information about contaminants that could make them sick would affect their handling of tree nuts more than information about contaminants that could be lethal (47%; *n* = 981), followed by scientific research on microbial food safety risks associated with tree nuts (39%; *n* = 981) (Table S12). Government websites (52%; *n* = 981) constituted the most preferred source for the delivery of tree nut food safety information, followed by company websites (47%; *n* = 981) and social media (45%; *n* = 981) (Figure 6).

### 3.6. Relationship between Knowledge, Perceptions, and Handling Practices

A two-step cluster analysis was generated for the nine knowledge and four perception statements about tree nuts (Table 3). In eight of the nine knowledge statements and all perception statements, Cluster 1 “low knowledge and perception” had a lower mean than Cluster 2 “high knowledge and perception,” which suggests that the participants in Cluster 1 had lower knowledge and perceptions than the participants in Cluster 2 (Table 3). The one knowledge statement that Cluster 1 reported to have a higher mean than Cluster 2 was “All tree nuts are free of harmful bacteria.” Cluster 1 “low knowledge and perception” mostly represented participants (*n* = 373) who were between the ages of 18 and 24 or 45 and above, female, white or Hispanic, from a suburban or rural community, held an associate degree or lower, earned an annual household income less than USD 100,000, and followed no specific diet. Cluster 2 “high knowledge and perception” mainly represented participants (*n* = 98) who were between the ages of 25 and 44, male, non-white or Hispanic, from an urban community, held a bachelor’s degree or more advanced degree, earned an annual household income of USD 100,000 and above, and followed specific diets. In seven of the nine knowledge statements, a significant difference was exhibited between Cluster 1 “low knowledge and perception” and Cluster 2 “high knowledge and perception.” The difference was significant between the clusters for all demographic groups except ethnicity (*p* = 0.218).

Table 4 shows the Pearson correlation coefficients between handling practices and the four knowledge variables and three risk perception variables. A positive correlation indicates that safe handling practices are associated with high knowledge and perceptions, while a negative correlation suggests that safe handling practices are associated with low knowledge or perceptions. The behavior of adding salt or acid to the soaking water had the highest positive correlation observed with the knowledge of tree nut foodborne outbreaks and recalls (r = 0.25; *p* < 0.01). Also, the behaviors of adding salt or acid to the soaking water and drying soaked nuts for 12 h or more had the highest positive correlation with the perception of contracting a foodborne illness from consuming tree nuts (r = 0.14; *p* < 0.01).

## 4. Discussion

Consumers’ food safety knowledge and perception of tree nuts can influence their handling practices. The present study is the first of its kind to analyze how food safety knowledge, perception of the risks and benefits of tree nuts, and preferred sources for food safety information influences consumers’ handling practices of tree nuts at home. The findings of this study fill the data gaps in understanding consumer tree nut handling at home.

### 4.1. Tree Nut Food Safety Knowledge

Not all consumers were knowledgeable about the potential contamination of tree nuts. While many participants (64%) acknowledged that harmful bacteria could survive on tree nuts for a long period of time, only a portion (35%) deduced that harmful bacteria may survive on the tree nuts they purchase. Although Salmonella cannot grow in low-moisture foods, it is known to survive for a long period of time [9]. Previous research has isolated foodborne pathogens, primarily Salmonella, from 0.35 to 4.20% of 375 g U.S. retail samples of raw cashews, hazelnuts, macadamia nuts, pecans, pine nuts, pistachios, and walnuts [12,13]. Only 69% of the survey participants recognized that tree nuts can be contaminated during harvest and processing events. Previous consumer surveys also conveyed that consumers possess inadequate knowledge about the contamination of nuts and other low-moisture foods [24,37]. Most tree nuts are allowed to fall naturally or are mechanically shaken to the ground, and then left for 7–10 days to dry in the sun [38], and the exposure to soil, water, animals, and insects may provide opportunities for potential contamination [39,40]. After harvesting, tree nuts also may become contaminated during processing, distribution, and consumer handling [39].

In the present study, few consumers (25%) were aware of any tree nut-related outbreaks and recalls. This finding aligns with a previous survey that found that 24% of survey participants were aware of foodborne illnesses associated with raw tree nuts [24]. While that study was conducted in 2011, several outbreaks linked to tree nuts had occurred since 2000 [14]. Additionally, similar results have been found with other low-moisture foods. Previous surveys reported that only 15% of consumers were aware of flour recalls [21], and only 22% of pet owners had heard of foodborne outbreaks or recalls associated with dried pet food [22]. Also, consumers lacked knowledge of treatment methods that could or could not eliminate harmful bacteria. In the present study, only a third (35%) of participants knew that freezing does not kill harmful bacteria. While freezing generally inhibits pathogen growth on food, it does not cause pathogen inactivation or death [41]. The misconception of freezing is comparable to another consumer survey, in which 42% of participants believed that freezing kills microorganisms [42].

### 4.2. Perception of Tree Nut Risks

Statistically significantly more participants perceived themselves to be likely to contract a foodborne illness from tree nuts in comparison to the number of participants who considered themselves at high risk for foodborne illness in general. While tree nuts are generally perceived as a low-risk food compared to other foods, such as animal products and produce [23,43], the contradicting finding from the present study may be due to the language used; that is, consumers may be more apprehensive to say they are “likely” as opposed to “at high risk” of contracting a foodborne illness since the average consumer is not at high risk. Also, consumers may perceive tree nuts as presenting a lower risk than other foods of conveying foodborne illness because of greater public consciousness about animal products and produce food safety risks. Additionally, tree nuts are a low-moisture food, and consumers do not associate many dry foods with microbial risks. A study analyzing dried pet food found that only 25% of pet owners believed that their pet food posed a food safety risk [22]. Furthermore, many consumers hold an “optimism bias,” a belief that they are less likely than the average person to experience a negative event [44]. In a consumer survey on food recalls, 38% of respondents indicated they are less likely than other Americans to believe that the foods they purchase could be recalled [45]. Another study found that young adults hold an optimism bias around food safety because they believe that their food preparation skills will help prevent a foodborne illness [30]. Optimism bias poses a food safety concern, because a low-risk perception may deter consumers from applying prudent and safe tree nut handling practices at home. According to the Health Belief Model, consumers with lower risk perceptions are less likely than more cautious consumers to engage in safe practices, such as hand washing or sanitizing when handling foods perceived as less risky [26].

### 4.3. Perception of Health Benefits

Many consumers emphasized that they prepared soaked nuts and NBDAs at home for the health benefits. Similarly, previous consumer surveys have reported that consumers most commonly perceive plant-based diets to be healthier than no specific diet [46,47]. Also, a content analysis of soaked nuts and NBDA recipes indicated that consumers claim that these tree nut products have an array of health benefits [11]. In the present study, the consumers perceived soaked nuts and NBDAs prepared at home as fresh, flavorful, with controlled ingredients, and free of preservatives or additives. This reasoning may also be related to health because in the “2023 Food and Health Survey”, consumers reported the top definition of healthy foods to be “fresh” [46]. Consumers have also been shown to gauge healthiness based on the nutritional content, ingredients, degree of processing, and use of hormones, antibiotics, or preservatives [46,48,49]. Overall, consumers of plant-based products tend to place a high importance on health [50], and believe foods considered “healthy” should be included more into the diet [48].

Furthermore, many consumers regard homemade foods as healthier than commercially produced foods [51]. Previous studies found that people who cook at home eat healthier by consuming less sugar, fats, carbohydrates, and calories and more vitamins to maintain a normal body weight and reduce the risk of diabetes [52,53]. Nut-based products have been linked to being high in protein, vitamins, and minerals, and low in saturated fats and cholesterol, which collectively may reduce the risk of cardiovascular disease, inflammation, and oxidative stress [54,55,56]. Additionally, soaking almonds overnight has been shown to enrich the vitamin E content to improve memory impairments when consumed on an empty stomach [57]. However, not all perceived health benefits are supported with scientific evidence, and some studies actually disproved consumer claims. For example, 38% of participants claimed that soaking nuts removes phytic acid, an enzyme inhibitor present in tree nuts, and 31% claimed that soaking nuts aids in the digestion of nutrients. Yet, previous studies found little evidence of a significant decrease in phytic acid in soaked almonds, and no improvement in the absorption and digestion of nutrients [58,59].

Consumers perceive raw tree nuts as free of preservatives, additives, and chemicals, and that they maintain their nutrients and taste better than processed nuts, and more than a third (35%) of participants preferred using raw tree nuts, which corroborates the findings from previous studies [7,11,24]. Research has found that consumers tend to be more concerned with toxic chemicals and pesticides than they are with microorganisms [42]. However, many participants in the present study were unaware that nuts labeled “raw” may be pasteurized (48%), and that pasteurization maintains raw characteristics (49%). At the time that study was conducted, no studies had produced evidence that raw tree nuts have a significantly lower risk of toxic chemical contamination nor higher nutritional value than pasteurized tree nuts. Current pasteurization techniques for raw almonds, which consists of blanching, roasting, using gas propylene oxide (PPO), or steam treatment to kill harmful pathogens, have been shown to prolong shelf life without altering nutrition or flavor [60]. While all commercially sold “raw” almonds in the United States require pasteurization, other tree nuts are not upheld to the same standard [61,62]. Thus, consumers may not know whether the tree nuts they purchased have been treated to reduce foodborne pathogens. Foodborne pathogens, primarily Salmonella, have been isolated from raw tree nuts purchased at retail stores [12,13], and many consumers source their tree nuts from retail channels [24,35].

### 4.4. Perception of Information Sources

Consumers were asked who in the food supply chain they felt were responsible for tree nut-related outbreaks or recalls, and who they would continue to trust following an outbreak or recall: the farmers, processors, distributors, supermarkets, and/or the government. While each food supply chain entity was given some responsibility, 71% of participants said they would suspect supermarkets and 70% would suspect processors as responsible in the case of a tree nut-related outbreak. Similarly, in another consumer survey, consumers ranked food processors as the most important food supply chain entity responsible for the food safety of animal products [43]. A literature review found that trust in food manufacturers and retailers was directly and positively related to food safety perceptions, because consumers perceived these entities to be primarily responsible for the safety of processed foods [63].

The consumers responded that they would continue to trust all entities in the food supply chain following a tree nut outbreak or recall. Consumers with lower risk perceptions about food safety, such as the participants in the present study, were more likely to trust entities in the food supply chain [64]. The farmers and distributors were shown to be the most trustworthy to consumers (both 45%). This finding contradicted a previous consumer survey that found participants blamed farmers the most for a multistate outbreak in produce [65]. The difference in responses between surveys may be due to consumers being more aware of potential food safety risks associated with produce.

Surprisingly, only 39% of consumers reported that they trust government assessments following a tree nut-related outbreak, which suggested that government agencies were the least trustworthy entity. This statistic contradicted other consumer surveys that found a higher degree of trust in government agencies to ensure food safety [46,66]. In fact, when consumers were asked for their preferred source for tree nut food safety information, the government was their top response. This reinforces previous studies that government agencies are the preferred sources for food safety information [33,67]. Also, consumers reported that they prefer company websites and social media for tree nut food safety information. A recent consumer survey found that 42% of participants viewed food and nutrient content from social media [46]. Additionally, many consumers have acknowledged obtaining recipes for tree nut products from social media [35].

### 4.5. Factors Influencing Consumer Knowledge, Perceptions, and Handling Practices

Many factors, including age, gender, ethnicity, level of education, and income, can influence a consumer’s knowledge, perception, and behavior. According to the cluster analysis, the consumers who were less knowledgeable about food safety and held lower risk perceptions tended to use risky behavior when handling tree nuts. The correlation between low food safety knowledge and risk perception, and unsafe handling practices aligns with previous research [16,17]. The demographic groups with lower food safety knowledge and risk perceptions were between the ages of 18 and 24 or 45 and above, female, white or Hispanic, from suburban and rural communities, held an associate degree or lower, and earned an annual household income of less than USD 100,000. Insufficient knowledge and failure to perceive potential risks can contribute to outbreaks of foodborne illnesses in the home [68].

The finding that people of ages 18 to 24 or 45 and above had low knowledge and risk perception is consistent with previous studies. A meta-analysis showed that young adults were the least knowledgeable about safe handling behavior [19]. Other surveys reported that food safety knowledge in young adults has been shown to be typically low due to reliance on family and friends rather than educational materials as sources of food safety information [16,30,31]. Similarly, some older adults who may be knowledgeable about food safety behavior do not necessarily partake in appropriate food safety behavior to reduce the risk of foodborne illness [69,70].

The consumers with a lower income had lower food safety knowledge and risk perception than the high-income consumers. Similar findings have reported that low-income individuals lack food safety knowledge and have lower levels of safe handling behaviors, like handwashing during food preparation [34,71,72,73]. Additionally, consumers with lower incomes are less likely to purchase tree nuts [1] and may be less concerned about tree nut food safety.

In the present study, the consumers who held an associate degree or lower had lower food safety knowledge and risk perception. Consumer knowledge, perception, and handling behavior based on education level varied compared with previous research. Consumers with less education have been reported to have less knowledge of food safety and to lack safe handling behaviors, which aligns with the present study [18,19].

The findings that females in suburban and rural communities had lower knowledge and perceptions and practice unsafe handling behaviors contradicts previous studies. Females often display more food safety knowledge and safer handling behavior than males [16,17,18,31]. Consumers in metropolitan areas have been reported to engage in behavior that is more risky than that of nonmetropolitan consumers [19].

## 5. Limitations

Although this study was carefully designed, it has some limitations. Due to the restricted size of the targeted audience and the tendency for more women and younger generations to soak tree nuts or make products from them, the participant distribution did not match the U.S. census data, and the results may not be representative of the U.S. population. Also, the results may reflect a self-reporting bias; that is, what people say they do may differ from what they actually do because of social desirability or difficulty in recalling past events [74]. This may lead to skewed results in the cluster analysis to identify influencing factors for consumer knowledge, risk perception, and behavior. Furthermore, the participants were paid for their time and contribution, which can be an incentive for them to complete the survey even if it does not apply to them. Nevertheless, the self-reported information on tree nut soaking and NBDAs sheds light on understanding the food safety implications of tree nut handling.

## 6. Conclusions

This study examined consumers’ knowledge and perceptions of the risks and benefits of tree nut handling and identified the factors that influence consumer knowledge and perception of and behaviors in tree nut handling. The survey results revealed that many consumers displayed somewhat low food safety knowledge on the potential contamination of tree nuts. Most consumers were unaware of tree nut-related outbreaks and recalls, and many expressed confusion about raw tree nuts and tree nut pasteurization. Significantly more participants perceived themselves to be likely to contract a foodborne illness from tree nuts than from other foods in general. Additionally, consumers considered the health benefits of tree nuts, especially when making soaked nuts and NBDAs, more than the food safety risk. When asked who to trust for tree nut food safety information, the government was the top preferred source. The cluster analysis showed that the demographic groups with lower food safety knowledge and risk perceptions were between the ages of 18 and 24, or 45 and above, female, white or Hispanic, from suburban and rural communities, held an associate degree or lower, and earned an annual household income less than USD 100,000. Lastly, consumers who had lower knowledge and risk perception tended to practice more risky handling behaviors with tree nuts. The findings highlighted the importance of understanding the food safety implications and influencing factors of consumer tree nut handling behavior, which can help inform future consumer food safety education research and in designing audience-targeted educational materials for tree nut food safety.

## Figures and Tables

**Table 1:** Demographic groups and knowledge and perception clusters: Chi-square tests.

Demographic Group	No. (%) of Responses (*n* = 981)	Clusters			
		Low Knowledge and Perception (*n* = 829)	High Knowledge and Perception (*n* = 138)	*X*^2^ (df)	*p*
Age					
18–24	156 (15.9)	152 (18.3)	4 (2.9)		
25–34	329 (33.5)	275 (33.2)	51 (37.0)		
35–44	240 (24.5)	265 (32.0)	69 (50.0)		
45–54	118 (12.0)	106 (12.8)	9 (6.5)		
55–64	87 (8.9)	85 (10.3)	2 (1.4)		
65 or above	47 (4.8)	42 (5.1)	3 (2.2)		
Prefer not to answer	4 (0.4)	4 (0.5)	NA ^a^	79.2 (6)	<0.001
Gender					
Female	612 (62.4)	565 (68.2)	40 (29.0)		
Male	359 (36.6)	254 (30.6)	98 (71.0)		
Prefer not to answer	10 (1.0)	10 (1.2)	NA	83.7 (2)	<0.001
Ethnicity					
White (non-Hispanic)	659 (67.2)	557 (67.2)	93 (67.4)		
African American	130 (13.3)	104 (12.5)	23 (16.7)		
Hispanic	95 (9.7)	89 (10.7)	7 (5.1)		
Asian or Pacific Islander	61 (6.2)	47 (5.7)	11 (8.0)		
Native American	13 (1.3)	12 (1.4)	1 (0.7)		
Other	16 (1.6)	13 (1.6)	7 (5.1)		
Prefer not to answer	7 (0.7)	3 (0.4)	NA	8.3 (6)	0.218
Community					
Suburban	417 (42.5)	395 (47.6)	18 (13.0)		
Urban	354 (36.1)	236 (28.5)	112 (81.2)		
Rural	210 (21.4)	198 (23.9)	8 (5.8)	142.6 (2)	<0.001
Education level					
Less than high school (HS) diploma	22 (2.2)	20 (2.4)	1 (0.7)		
HS diploma/GED ^b^	178 (18.1)	174 (21.0)	8 (5.8)		
Some college (no degree)	232 (23.6)	220 (26.5)	5 (3.6)		
Associate degree	118 (12.0)	111 (13.4)	7 (5.1)		
Bachelor’s degree	266 (27.1)	185 (22.3)	74 (53.6)		
Graduate degree	159 (16.2)	113 (13.6)	43 (31.2)		
Prefer not to answer	6 (0.6)	6 (0.7)	NA	116.3 (6)	<0.001
Household income					
Less than USD 25,000	132 (13.5)	119 (14.4)	11 (8.0)		
USD 25,000–USD 49,999	240 (24.5)	230 (27.7)	8 (5.8)		
USD 50,000–USD 74,999	219 (22.3)	202 (24.4)	18 (13.0)		
USD 75,000–USD 99,999	170 (17.3)	135 (16.3)	31 (22.5)		
USD 100,000–USD 149,999	116 (11.8)	77 (9.3)	37 (26.8)		
USD 150,000–USD 199,999	50 (5.1)	30 (3.6)	18 (13.0)		
USD 200,000 and above	26 (2.7)	9 (1.1)	15 (10.9)		
Prefer not to answer	28 (2.9)	27 (3.3)	NA	138.1	<0.001
Diet					
No specific diet	511 (51.1)	460 (55.5)	44 (31.9)		
Dairy-free	126 (12.8)	101 (12.2)	21 (15.2)		
Vegetarian	106 (10.8)	77 (9.3)	27 (19.6)		
Keto	66 (6.7)	61 (7.4)	5 (3.6)		
Vegan	65 (6.6)	47 (5.7)	18 (13.0)		
Gluten-free	42 (4.3)	29 (3.5)	11 (8.0)		
Whole30	23 (2.3)	19 (2.3)	4 (2.9)		
Paleo	18 (1.8)	12 (1.4)	7 (5.1)		
Other	24 (2.4)	23 (2.8)	1 (0.7)	52.9	<0.001

Note: 14 responses were excluded from the cluster distribution. ^a^ NA: not applicable. ^b^ GED: general education development.

**Table 2:** Participants’ perception of raw and pasteurized tree nuts before and after reading a food safety message: Chi-square tests.

	No. (%) of Responses (*n* = 981)		*X*^2^ (df)	*p*
	Before	After		
Raw tree nuts have more of a health benefit than treated tree nuts	160 (16.3)	228 (23.2)	541 (16)	<0.001
The health benefits of raw tree nuts are more important than the microbial risk	233 (23.8)	301 (30.7)	744 (16)	<0.001

**Table 3:** Knowledge and perception clusters and means of knowledge and perception statements: one-way ANOVAs according to Bonferroni tests.

Knowledge or Perception Statement	Clusters		*F*	*p*
	Low Knowledge and Perception (*n* = 829)	High Knowledge and Perception (*n =* 138)		
**Knowledge (range: 1–3)**				
Tree nuts can be contaminated with harmful bacteria	2.42	2.82	17.0	<0.001
Pasteurization processes can maintain raw characteristics	2.25	2.72	30.8	<0.001
Raw tree nuts have a higher risk for contamination	2.23	2.65	23.7	<0.001
Harvesting and processing provides opportunities for contamination	2.15	2.57	14.7	<0.001
Harmful bacteria can survive on tree nuts for long periods of time	2.09	2.29	3.1	0.079
All tree nuts are free of harmful bacteria ^a^	1.73	1.54	4.7	0.030
Some tree nuts labeled as “raw” have been pasteurized	1.70	2.62	79.5	<0.001
Foodborne outbreaks or recalls have been associated with tree nuts	1.39	2.26	132.9	<0.001
Freezing destroys any harmful bacteria on tree nuts ^a^	1.33	1.52	2.8	0.093
**Perceptions (range: 1–5)**				
Tree nuts pose a high risk for microbial contamination	3.02	4.07	125.1	<0.001
Tree nuts that consumers purchase are likely to be contaminated	2.95	4.01	127.6	<0.001
Consumers are likely to contract a foodborne illness from tree nuts	2.70	3.92	145.1	<0.001
Tree nuts pose a high risk for contracting a foodborne illness	2.46	3.88	195.5	<0.001

^a^ Incorrect reverse-coded statements.

**Table 4:** Pearson correlation coefficient of knowledge, perception, and behavior.

		1	2	3	4	5	6	7	8
1	Knowledge: Tree nut outbreaks and recalls	--							
2	Perception: Foodborne illness from tree nuts	0.26 **	--						
3	Behavior: Washing hands with soap and water	0.07 *	0.09 **	--					
4	Behavior: Soak nuts for less than 8 h	0.16 **	0.08 *	0.19 **	--				
5	Behavior: Soak nuts in refrigerator	0.19 **	0.12 **	0.22 **	0.74 **	--			
6	Behavior: Add salt or acid to soaking water	0.25 **	0.14 **	0.21 **	0.68 **	0.78 **	--		
7	Behavior: Soak dry nuts for 12 h or more	0.19 **	0.14 **	0.17 **	0.37 **	0.35 **	0.35 **	--	
8	Behavior: Soak dry nuts at 165° F or higher	0.07	0.02	0.03	−0.06	−0.05	0.04	−0.09	--

* *p*-value < 0.05; ** *p*-value < 0.

## Data Availability

Data are contained within the article or Supplementary Material.

## Supplementary Materials

The following supporting information can be downloaded at: https://www.mdpi.com/article/10.3390/foods12234289/s1, Table S1: Participants’ sources for recipes for soaked nuts and nut-based dairy analogs (NBDA); Table S2: Participants’ knowledge on how tree nuts are grown and harvested; Table S3: Participants’ response to tree nut harvesting message; Table S4: Tree nuts involved in foodborne outbreaks or recalls that participants were aware of; Table S5: Participants’ perceptions of tree nut food safety risks; Table S6: Participants’ perceived raw characteristics of tree nuts lost during pasteurization; Table S7: Participants’ rationale for preferring raw over treated tree nuts; Table S8: Participants’ perceived benefits for soaking tree nuts for direct consumption and subsequent preparation of nut-based dairy analogs (NBDA); Table S9: Participants’ rationale for making nut-based dairy analogs (NBDA) at home rather than buying commercially and dairy-based products; Table S10: Participants’ method of consuming homemade nut-based dairy analogs (NBDA); Table S11: Participants’ actions taken when tree nuts they purchased have been recalled; Table S12: Participants’ reported information that would affect their handling of tree nuts.
