# Supplementary material for: U.S. Consumers’ Tree Nut Food Safety Knowledge, Perceptions, and Handling Practices across Demographic Groups
Source: Foods. 2023 Nov 28;12(23):4289. doi: 10.3390/foods12234289 (PMC10706404; doi:10.3390/foods12234289)
Supplement: Supplementary file 1 [file foods-12-04289-s001.zip › foods-2738083-supplementary.pdf]

Supplementary Materials

Table S1. Participants' sources for recipes for soaked nuts and nut-based dairy analogs (NBDA)

Source	Soaked nuts (n = 655)[a]	NBDA (n = 934)[a]
YouTube	388 (59.2)	546 (58.5)
Social media	341 (52.1)	487 (52.1)
Cookbooks	242 (36.9)	378 (40.5)
Friends/family	204 (31.1)	311 (33.3)
Blogs	168 (25.6)	256 (27.4)
Newspaper	117 (17.9)	124 (13.3)
Other[b]	24 (3.7)	40 (4.3)

[a] Due to multiple sources selected, calculated numbers and proportions exceed the actual number of participants.

[b] Other source mentioned were "web search," "Google," "websites," "Pinterest," "dietitian," and "through my own research."

Table S2. Participants' knowledge on how tree nuts are grown and harvested

Topic	Very knowledgeable (n = 981)	Somewhat knowledgeable (n = 981)	Not at all knowledgeable (n = 981)
Grown	225 (22.9)	463 (47.2)	293 (29.9)
Harvested	201 (20.5)	458 (46.7)	312 (21.8)

Table S3. Participants' response to tree nut harvesting message

Topic and information	No. (%) of responses (n = 981)
Surprising information[a]	194 (19.8)
Tree nuts left on ground to dry	450 (45.9)
Almonds and walnuts shaken mechanically to ground	274 (27.9)
Tree nuts dried outside	270 (27.5)
Tree nuts naturally fall to ground	312 (21.8)
Nothing surprising	194 (19.8)
Other[b]	6 (0.6)
Food safety risk[a]	
Contaminated with dust and other particles	625 (63.7)
Contaminated with foodborne pathogens	543 (55.4)
Contaminated with insects	424 (43.2)
Other[c]	647 (66.0)

[a] Due to multiple responses selected, calculated numbers and proportions exceed the actual number of participants.

[b] Other surprising information mentioned were "never heard of cashew apples" and "how cashews are harvested."

[c] Other food safety risks mentioned were "unknown risks," "pesticides," "ground contamination," "didn't know they could get contaminated," and "I don't know."

Table S4. Tree nuts involved in foodborne outbreaks or recalls that participants were aware of

Tree nut	No. (%) of responses (*n* = 247)[a]
Walnuts	124 (50.2)
Almonds	120 (48.6)
Cashews	114 (46.2)
Pistachios	83 (33.6)
Does not remember	30 (12.1)

[a] Due to multiple tree nuts selected, calculated numbers and proportions exceed the actual number of participants.

Table S5. Participants' perceptions of tree nut food safety risks

Perception	No. (%) of response (*n* = 981)		
	Agree	Neither Agree nor Disagree	Disagree
I am at high risk of contracting a foodborne illness	80 (8.2)	293 (29.7)	241 (24.6)
Tree nuts pose a high risk for microbial contamination	386 (39.3)	341 (35)	254 (25.9)
Tree nuts I purchase are likely to be contaminated	362 (36.9)	361 (36.8)	258 (26.3)
I am likely to contract a foodborne illness from consuming contaminated tree nuts	290 (29.6)	313 (31.9)	378 (38.5)
I am at high risk of contracting a foodborne illness	239 (24.4)	293 (29.7)	449 (45.8)

Table S6. Participants' perceived raw characteristics of tree nuts lost during pasteurization

Raw characteristic	No. (%) of responses (n = 981)[a]
Flavor	382 (38.9)
Source of un unsaturated fats, vitamins, and minerals	366 (37.3)
High protein and dietary fiber content	362 (36.9)
Freshness	362 (36.9)
All-natural characteristic	271 (27.6)
Being free of preservatives, additives, or chemical substances	262 (26.7)
Low saturated fat and cholesterol content	196 (20.0)
No health benefits lost	147 (15.0)
Other	16 (1.6)

[a] Due to multiple rationales selected, calculated numbers and proportions exceed the actual number of participants.

Table S7. Participants' rationale for preferring raw over treated tree nuts

Rationale	No. (%) of responses (n = 323)[a]
Free of preservatives, additives, or chemical substances	153 (47.4)
Nutrients intact	130 (40.2)
Flavor	104 (32.2)
Sustainable	72 (22.3)
Fair trade	59 (18.3)
Cost effective	55 (17.0)

[a] Due to multiple rationales selected, calculated numbers and proportions exceed the actual number of participants.

Table S8. Participants' perceived benefits for soaking tree nuts for direct consumption and subsequent preparation of nut-based dairy analogs (NBDA)

Benefit	No. (%) of responses (n = 718)[a]
Make nuts easier to eat or blend	365 (50.8)
Removes chemical substances	351 (48.9)
Removes harmful bacteria	343 (47.8)
Removes nutritional inhibitors	267 (37.2)
Improves taste	224 (31.2)
Aids digestions of nutrients	217 (30.2)
Recipe advises to do so	180 (25.1)
"Activates" the nuts	124 (17.3)
Other[b]	4 (0.6)

[a] Due to multiple rationales selected, calculated numbers and proportions exceed the actual number of participants.

[b] Other rationales for soaking mentioned were "taste," "to soften the nuts," "regimen recipe," "no reason," "allergic," "habit," "to clean the nuts," and "for a specific texture."

Table S9. Participants' rationale for making nut-based dairy analogs (NBDA) at home rather than buying commercially and dairy-based products

Rationales	No. (%) of responses (n = 934)[a]	
	Buying commercially	Dairy-based products
Healthier	508 (54.4)	595 (63.7)
Fresher	412 (42.0)	NA[b]
More flavorful	379 (40.6)	322 (34.5)
Control the ingredients	347 (37.2)	NA
Free of preservatives, additives, or chemical substances	312 (33.4)	NA
More cost effective	303 (32.4)	283 (30.3)
More sustainable	223 (23.9)	280 (30.0)
Enjoys the process	218 (23.3)	NA
Does not consume dairy	NA	115 (12.3)
Can use up extra tree nuts	88 (9.4)	NA
Sells (farmer's market, roadside stand, on farm stores)	69 (7.4)	NA
Other[c]	27 (2.9)	44 (4.7)

[a] Due to multiple rationales selected, calculated numbers and proportions exceed the actual number of participants.

[b] NA, not applicable.

[c] Other rationales to make NBDA at home mentioned were "wanted to try," "no reason," "medical reason," "lactose intolerance," "fun to try," and "don't need a lot."

Table S10. Participants' method of consuming homemade nut-based dairy analogs (NBDA)

Method of consuming	No. (%) of responses (n = 934)[a]
Directly as it is	547 (58.6)
In cereals or oatmeal	508 (54.4)
In baked products	451 (48.3)
In other dishes or recipes	410 (43.9)
Paired with other foods (i.e., crackers, bread, granola)	350 (37.5)
As a substitute for dairy products	326 (34.9)
Other[b]	561 (60.1)

[a] Due to multiple methods selected, calculated numbers and proportions exceed the actual number of participants.

[b] Other methods of consuming NBDA mentioned were for smoothies, protein shakes, coffee, cakes and cookies, and hot chocolate.

Table S11. Participants' actions taken when tree nuts they purchased have been recalled

Action	No. (%) of responses (*n* = 981)[a]
Throw away recalled nuts	731 (74.5)
Cook recalled nuts	124 (12.6)
Still eat recalled nuts	105 (10.7)
Feed recalled nuts to pets	50 (5.1)
Use recalled nuts for crafts	49 (5.0)
Other[b]	17 (1.7)

[a] Due to multiple tree nuts selected, calculated numbers and proportions exceed the actual number of participants.

[b] Other actions taken with recalled tree nuts mentioned were "return to store/retailer," "give to squirrels," "compost," and "depends on reason that they were recalled."

Table S12. Participants' reported information that would affect their handling of tree nuts

Information	No. (%) of responses (n = 981)[a]
Foodborne outbreaks that can make you sick	546 (55.7)
Foodborne outbreaks that can make you die	465 (47.4)
Scientific research about microbial food safety risk associated with tree nuts	379 (38.6)
Other[b]	3 (0.3)

[a] Due to multiple tree nuts selected, calculated numbers and proportions exceed the actual number of participants.

[b] Other information that would affect their tree nut handling mentioned were "I would just purchase from another source," "I trust my organic coop," and "I listen to my doctor."

Appendix

In this study, we will be asking you questions about your handling practices and food safety knowledge associated with tree nuts. For this study, 'tree nuts' refer to almonds, walnuts, cashews, and pistachio. The study focuses on handling practices when making nut-based dairy alternatives and soaked (sometimes referred as 'activated' or 'sprouted') nuts for direct consumption. This study defines 'nut-based dairy alternatives' as nut milk, cheese, and yogurt.

Block 0: Screening

1. Are you the primary shopper for yourself and/or your household?
   - o Yes
   - o No

   [End survey if consumer selected 'No']
2. How often do you use tree nuts to make nut-based dairy alternatives at home?
   - o Never
   - o Rarely (less than 2 times in the past year)
   - o Sometimes (2–12 times in the past year)
   - o Regularly (more than 12 times in the past year)
3. *[If they make nut-based products]* What are the tree nuts you use to make nut-based dairy alternatives at home? (Select all that apply)
   - ▫ Almonds
   - ▫ Walnuts
   - ▫ Cashews
   - ▫ Pistachios
   - ▫ Pecans
   - ▫ Peanuts
   - ▫ None of the above
4. How often do you soak tree nuts for direct consumption at home?
   - o Never
   - o Rarely (less than 2 times in the past year)
   - o Sometimes (2–12 times in the past year)
   - o Regularly (more than 12 times in the past year)
5. *[If they soak nuts for consumption only]* What are the tree nuts you soak for direct consumption at home? (Select all that apply)

- ▫ Almonds
- ▫ Walnuts
- ▫ Cashews
- ▫ Pistachios
- ▫ Pecans
- ▫ Peanuts
- ▫ None of the above

[End survey if consumer selected 'Never' or 'Rarely' for question 2 AND 4 or selected 'None of the above' for question 3 AND 5]

Block 2: Handling Tree Nut-Based Dairy Alternatives

[This block will only be displayed for consumers that selected 'Sometimes' or 'Regularly' in screening question 2]

In this section, we will ask questions about your handling of tree nuts when making nut-based dairy alternatives at home. For this study, 'nut-based dairy alternatives' refer to nut-based milk, cheese, and yogurt made from tree nuts and 'tree nuts' refer to almonds, walnuts, cashews, and pistachios.

1. *[If they soak all tree nuts the same]* Why do you soak your tree nuts? (select all that apply)
   - ▫ To remove nutritional inhibitors (e.g., tannin, phytic acid, or enzyme inhibitors)
   - ▫ To remove chemical substances (e.g., pesticides, preservatives, additives)
   - ▫ To remove harmful bacteria
   - ▫ To aid in digestion of nutrients
   - ▫ To make the nuts easier to blend
   - ▫ To sprout or activate the nuts
   - ▫ For a better taste of the final product
   - ▫ The recipe advises me to soak
   - ▫ Other, please specify: ____________________
2. *[If they soak each tree nut variety differently]* Why do you soak your tree nuts? (Select all that apply)

	**Almonds**	**Cashews**	**Walnuts**	**Pistachios**
To remove nutritional inhibitors (i.e., tannin, phytic acid, or enzyme inhibitors)	○	○	○	○
To remove chemical substances (i.e., pesticides, preservatives, additives)	○	○	○	○
To remove harmful bacteria	○	○	○	○
To aid in digestion of nutrients	○	○	○	○
To make the nuts easier to blend	○	○	○	○
To sprout or activate the nuts	○	○	○	○
For a better taste of the final product	○	○	○	○
The recipe advises to soak	○	○	○	○
I don't soak this nut	○	○	○	○
Other, please specify: ____________	○	○	○	○

3. *[If they remove the skin from their almonds]* Why do you remove the skins from your almonds? (Select all that apply)
   - ▫ To remove nutritional inhibitors within the skin (e.g., tannin or phytic acid)

- ▫ To remove chemical substances (e.g., pesticides, preservatives, additives)
- ▫ To remove harmful bacteria
- ▫ To aid in digestion of nutrients
- ▫ To create a smoother consistency for the final product
- ▫ To make the almonds easier to blend
- ▫ For a better taste of the final product
- ▫ The recipe advises to remove the skin
- ▫ Other, please specify: ____________________

4. *[If they strain their nut-based dairy alternatives]* What do you do with the leftover pulp (the by-product from making nut milk)? (Select all that apply)
   - ▫ Discard it
   - ▫ Compost it
   - ▫ Make nut flour
   - ▫ Use in other recipes
   - ▫ Other, please specify: ______________________
5. Where do you find recipes to make nut-based dairy alternatives at home? (Select all that apply)
   - ▫ Blog
   - ▫ Newspaper
   - ▫ YouTube
   - ▫ Social media (e.g., Instagram, TikTok, Facebook, Twitter, etc.)
   - ▫ Cookbooks
   - ▫ Friends/Family
   - ▫ Other, please specify: ______________________
6. Why do you make nut-based dairy alternatives at home than buying commercially? (Select all that apply)
   - ▫ Homemade is healthier than the store bought
   - ▫ Homemade is more flavorful than store bought
   - ▫ Homemade is fresher than store bought
   - ▫ Homemade is more cost effective than store bought
   - ▫ Homemade is more sustainable than store bought
   - ▫ Homemade is free of preservatives, additives, and chemical substances
   - ▫ I can control and know what ingredients are going into the products
   - ▫ I sell my homemade products (i.e., farmer's market, roadside stands, on farm stores)
   - ▫ I enjoy the process of making homemade nut-based products
   - ▫ I can use up extra tree nuts
   - ▫ Other, please specify: ______________________
7. Why do you make nut-based dairy alternatives at home rather than dairy-based products? (Select all that apply)
   - ▫ Nut-based is healthier
   - ▫ Nut-based is easier to make
   - ▫ Nut-based is more cost effective
   - ▫ Nut-based is more sustainable
   - ▫ Nut-based tastes better
   - ▫ I do not consume dairy products

- ▫ Other, please specify: ______________________

8. *[If they do not consume dairy products]* Why do you not consume dairy-based products? (Select all that apply)
   - ▫ Health reasons (i.e., dairy products are higher in saturated fats, cholesterol, and sugar)
   - ▫ Lactose intolerant or sensitive
   - ▫ Do not like the taste
   - ▫ Want to lose weight
   - ▫ Want to avoid animal products
   - ▫ Prefer plant-based products
   - ▫ Other, please specify: ______________________
9. How do you use your homemade nut-based dairy alternatives? (Select all that apply)
   - ▫ Directly as it is
   - ▫ In baked products
   - ▫ In other dishes or recipes
   - ▫ In cereals or oatmeal
   - ▫ Paired with other foods (i.e., crackers, bread, granola)
   - ▫ As substitute to dairy products
   - ▫ Other, please specify: ______________________

Block 3: Handling Soaked Nuts for Consumption

[This block will only be displayed if consumers selected 'Sometimes' or Regularly' in screening question 4].

In this section, we will ask questions about your handling practices when soaking (sometimes referred at 'activating' or 'sprouting') tree nuts for consumption use only. Again, in this study, 'tree nuts' refer to almonds, walnuts, cashews, and pistachios.

1. *[If they soak all tree nuts the same]* Why do you soak your almonds for direct consumption? (Select all that apply)
   - ▫ To remove nutritional inhibitors (e.g., tannin, phytic acid, or enzyme inhibitors)
   - ▫ To remove chemical substances (e.g., pesticides, preservatives, additives)
   - ▫ To remove harmful bacteria
   - ▫ To aid in digestion of nutrients
   - ▫ To make the nuts easier to eat
   - ▫ For a better taste
   - ▫ Other, please specify: ______________________
2. *[If they soak each tree nut variety differently]* Why do you soak your tree nuts for direct consumption? (Select all that apply)

	**Almonds**	**Cashews**	**Walnuts**	**Pistachios**
To remove nutritional inhibitors (i.e., tannin, phytic acid, or enzyme inhibitors)	○	○	○	○
To remove chemical substances (i.e., pesticides, preservatives, additives)	○	○	○	○
To remove harmful bacteria	○	○	○	○
To aid in digestion of nutrients	○	○	○	○
To make the nuts easier to blend	○	○	○	○
To sprout or activate the nuts	○	○	○	○
For a better taste	○	○	○	○

The recipe advises to soak	○	○	○	○
I do not soak this nut	○	○	○	○
Other, please specify: ____________	○	○	○	○

3. *[If they remove the skin from their almonds]* Why do you remove the skin from your almonds? (Select all that apply)
   - ▫ To remove nutritional inhibitors (i.e., tannin or phytic acid)
   - ▫ To remove chemical substances (i.e., pesticides, preservatives, additives)
   - ▫ To remove harmful bacteria
   - ▫ To aid in digestion of nutrients
   - ▫ To make the almonds easier to eat
   - ▫ For a better taste
   - ▫ Other, please specify: ____________________
4. Where do you find recipes to soak tree nuts for direct consumption? (Select all that apply)
   - ▫ Blog
   - ▫ Newspaper
   - ▫ YouTube
   - ▫ Social media (e.g., Instagram, TikTok, Facebook, Twitter, etc.)
   - ▫ Cookbooks
   - ▫ Friends/Family
   - ▫ Other, please specify: _____________________

Block 4: Raw Tree Nuts

[Question 1 is only displayed if the consumer selected 'Sometimes' or 'Regularly' in screening question 2]

1. When making nut-based dairy alternatives at home, what kind of tree nuts do you prefer to use?
   - o Roasted
   - o Blanched (tree nuts have been briefly boiled to remove the skins)
   - o Pasteurized (exposed to heat to remove pathogens)
   - o Depends, please specify: ____________________

[Question 2 is only displayed if the consumer selected 'Sometimes' or 'Regularly' in screening question 4]

1. When soaking nuts for direct consumption, what kind of tree nuts do you prefer to use?
   - o Roasted
   - o Blanched (tree nuts have been briefly boiled to remove the skins)
   - o Pasteurized (exposed to heat to remove pathogens)
   - o Depends, please specify: _____________________
2. *[If they prefer to use raw tree nuts]* For the following question, rate how important each factor is in choosing raw over treated tree nuts from "Not at all important" to "Extremely important".

	Not at all Important	Slightly Important	Moderately Important	Very Important	Extremely Important
Cost Effective	○	○	○	○	○
Sustainable	○	○	○	○	○
Flavor	○	○	○	○	○

Free of preservatives, additives, or chemical substances	○	○	○	○	○
Fair trading (agreement between trading entities to pay fair prices to achieve equitable trade relationships)	○	○	○	○	○

Pasteurization refers to exposure to heat to destroy pathogens in foods and beverages.

3. Are you aware that there are pasteurization processes that maintain the raw characteristics of tree nuts?
   - ○ Yes
   - ○ No
   - ○ I don't know
4. Are you aware that some tree nuts labeled as raw have been pasteurized?
   - ○ Yes
   - ○ No
   - ○ I don't know
5. Are you aware that raw (not roasted, blanched, pasteurized) tree nuts have a higher risk for contamination?
   - ○ Yes
   - ○ No
   - ○ I don't know
6. For the following question, please rate how strongly you disagree or agree with the following statement from "Strongly disagree" to "Strongly agree"

	**Strongly Disagree**	**Somewhat Disagree**	**Neither Agree nor Disagree**	**Somewhat Agree**	**Strongly Agree**
Raw, unpasteurized tree nuts have more of a health benefit than treated tree nuts	○	○	○	○	○

7. What health benefits do you believe are lost during pasteurization? (Select all that apply)
   - ▫ Low saturated and cholesterol content
   - ▫ High protein and dietary fiber content
   - ▫ Source of unsaturated fats, vitamin E, vitamin B-6, potassium, phosphorus, or magnesium
   - ▫ Flavor
   - ▫ Freshness
   - ▫ Being free of preservatives, additives, or chemical substances
   - ▫ All natural
   - ▫ I do not believe any health benefits are lost during treatment
   - ▫ Other, please specify: ______________________

8. For the following question, please rate how strongly you disagree or agree with the following statement from “Strongly disagree” to “Strongly agree”

	Strongly Disagree	Somewhat Disagree	Neither Agree nor Disagree	Somewhat Agree	Strongly Disagree
The health benefits of raw, unpasteurized tree nuts are more important than the microbial risks	○	○	○	○	○

9. Pathogens, such as *Salmonella,* can survive for sufficient time on low-moisture foods, such as tree nuts, to cause foodborne illness. *Salmonella* infections can lead to diarrhea, fever, and abdominal cramps and for the immunocompromised, elderly, and young children can cause a much more serious infection.

After reading the food safety message above, rate how strongly you disagree or agree with the following statement from “Strongly disagree” to “Strongly agree.”

	Strongly Disagree	Somewhat Disagree	Neither Agree nor Disagree	Somewhat Agree	Strongly Agree
Raw, unpasteurized tree nuts have more of a health benefit than treated tree nuts	○	○	○	○	○
The health benefits of raw, unpasteurized tree nuts are more important than the microbial risks	○	○	○	○	○

Block 5: Tree Nut Food Safety Knowledge and Risk Perception

1. For the following question, please indicate if you believe the following statements are true or false.

	True	False	I Don’t Know
Tree nuts can be contaminated with harmful bacteria	○	○	○
Harmful bacteria can survive on tree nuts for a long period of time	○	○	○
Harvesting and processing provides opportunities for cross contamination	○	○	○
Freezing will destroy any harmful bacteria on tree nuts	○	○	○

2. Do you expect the tree nuts you purchase to be free of harmful bacteria?
   - ○ Yes
   - ○ No

- ○ I don’t know

3. For the following question, please rate how strongly you disagree or agree with each of the following statements from “Strongly disagree” to “Strongly agree.”

	**Strongly Disagree**	**Somewhat Disagree**	**Neither Agree nor Disagree**	**Somewhat Agree**	**Strongly Agree**
I am at a high-risk level for contracting a foodborne illness.	○	○	○	○	○
Tree nuts pose a high-risk for microbial contamination.	○	○	○	○	○
The tree nuts I purchase are likely to be contaminated.	○	○	○	○	○
I am likely to contract a foodborne illness from consuming contaminated tree nuts.	○	○	○	○	○

4. Have you heard of recent tree nut related foodborne illness outbreaks or recalls?
   - ○ Yes
   - ○ No
   - ○ I don’t know
5. *[If they have heard of recent outbreaks or recalls]* What tree nuts were associated with the foodborne outbreak(s) or recall(s) that you heard about? (Select all that apply)
   - ▫ Almonds
   - ▫ Walnuts
   - ▫ Cashews
   - ▫ Pistachios
   - ▫ I do not remember
6. What information regarding tree nut related foodborne illness outbreaks or recalls would affect your handling or purchase of tree nuts? (Select all that apply)
   - ▫ Foodborne outbreaks that can make you sick
   - ▫ Foodborne outbreaks that can make you die
   - ▫ Scientific research about the microbial food safety risk associated with tree nuts
   - ▫ I don’t know
   - ▫ Other, please specify: ______________________
7. Would you throw away the tree nuts you purchased that were recalled?
   - ○ Yes, I would throw them away
   - ○ No, I would still eat them as is
   - ○ No, I would cook them
   - ○ No, I would feed them to my pets
   - ○ No, I would use them for crafts
   - ○ Other, please specify: ______________________

8. For the following question, please rate how strongly you disagree or agree that each of the following entities are responsible for a tree nut related foodborne outbreak and recall from “Strongly disagree” to “Strongly agree.”

	**Strongly Disagree**	**Somewhat Disagree**	**No Opinion**	**Somewhat Agree**	**Strongly Agree**
The farmer	○	○	○	○	○
The processor	○	○	○	○	○
The company	○	○	○	○	○
The supermarket (i.e., Meijer, Aldi, Costco)	○	○	○	○	○
The government (e.g., FDA, USDA, CDC)	○	○	○	○	○

9. For the following question, please rate how much trust you would continue to have with each of the following entities following a tree nut related foodborne outbreak and recall from “Strongly distrust” to “Strongly trust”

	**Strongly Distrust**	**Somewhat Distrust**	**No Opinion**	**Somewhat Trust**	**Strongly Trust**
The company	○	○	○	○	○
The processors	○	○	○	○	○
The farmers	○	○	○	○	○
The supermarkets (e.g., Meijer, Aldi, Costco)	○	○	○	○	○
The government (e.g., FDA, USDA, CDC)	○	○	○	○	○

10. How knowledgeable do you think you are about how tree nuts are grown and harvested?

	**Very Knowledgeable**	**Somewhat Knowledgeable**	**Not at all Knowledgeable**
Grown	○	○	○
Harvested	○	○	○

11. Tree nuts are grown on trees. After the fruits ripen, the tree nuts, like almonds and walnuts, fall to the groun either naturally or by mechanical shakers. Walnuts are usually collected soon after shaking and them dried at commercial dehydrators. Almonds are often dried on the ground before collection. Cashews are separated from fallen or harvested cashew apples, the fruit from which they are grown, and then dried in the sun. After drying, the tree nuts may be stored under cool conditions before they are processed and packaged.

After reading the statement, what information surprises you? (Select all that apply)

- ▫ Tree nuts are dried outside
- ▫ Tree nuts are left on the ground to dry
- ▫ Tree nuts fall natural to the ground
- ▫ Almonds and walnuts are shaken mechanically to the ground
- ▫ Nothing
- ▫ Other, please specify: ___________________

12. After reading the statement, what do you believe is the food safety risk associated with raw, unpasteurized tree nuts? (Select all that apply)
    - Contaminated with foodborne pathogens
    - Contaminated with dust and other particles
    - Contaminated with insects
    - Other, please specify: _________________

Block 6: Delivery Format of the Information

1. How would you like to learn more about tree nut food safety? (Select all that apply)
    - Brochure
    - Government websites (e.g., FDA, USDA, CDC)
    - Nut company website
    - Social Media (e.g., Instagram, TikTok, Facebook, Twitter, etc.)
    - TV
    - Newspapers
    - Word of mouth
    - Other, please specify: ______________________

Demographic Questions

1. What is your age?
    - 18–24
    - 25–34
    - 35–44
    - 45–54
    - 55–64
    - 65 or above
    - Prefer not to answer
2. What gender do you identify with the most?
    - Female
    - Male
    - Prefer not to answer
3. What ethnicity do you identify with the most? (Select all that apply)
    - White (Non-Hispanic)
    - Hispanic
    - African American
    - Asian or Pacific Islander
    - Native American
    - Other, please specify: ____________________
    - Prefer not to answer
4. Which of the following best describes the general area you live in?
    - Rural
    - Urban
    - Suburban
5. What state do you currently reside in?
    - Drop list of states
6. What is the highest degree or level of education you have completed?
    - Less than HS diploma
    - HS diploma/GED
    - Some college (no degree)
    - Associate degree

- Bachelor’s degree
- Graduate degree
- Prefer not to answer

7. Can you give us an estimate of your household income after taxes from last year?
   - Less than $25,000
   - $25,000–$49,999
   - $50,000–$74,999
   - $75,000–$99,999
   - $100,000–$149,999
   - $150,000–$199,999
   - $200,000 and above
   - Prefer not to answer
8. How would you describe your diet?
   - Vegan
   - Vegetarian
   - Keto
   - Paleo
   - Whole30
   - Dairy-free
   - Gluten-free
   - No specific diet
   - Other, please specify: ___________________
